# Effects of the COVID-19 pandemic on healthcare utilization among older adults with cardiovascular diseases and multimorbidity in Indonesia: an interrupted time-series analysis

**DOI:** 10.1186/s12889-023-17568-6

**Published:** 2024-01-02

**Authors:** Royasia Viki Ramadani, Mikael Svensson, Sven Hassler, Budi Hidayat, Nawi Ng

**Affiliations:** 1 School of Public Health and Community Medicine, Institute of Medicine, Sahlgrenska Academy, University of Gothenburg, Gothenburg, Sweden; 2 Center for Health Economics and Policy Studies, Faculty of Public Health, Universitas Indonesia, Jakarta, Indonesia; 3 Department of Pharmaceutical Outcomes & Policy, College of Pharmacy, University of Florida, Gainesville, USA; 4 Department of Health Sciences, Karlstad University, Karlstad, Sweden

**Keywords:** Chronic diseases, Multimorbidity, Covid, Healthcare utilization, Interrupted-time-series-analysis

## Abstract

**Background:**

The COVID-19 pandemic has disrupted healthcare utilization globally, but little is known about the effects among patients with cardiovascular diseases (CVDs) and other multimorbidities. This study analyzed the impacts of COVID-19 on healthcare utilization for patients aged 30 years and older with cardiovascular diseases (CVDs) with or without other chronic disease comorbidities in Indonesia.

**Methods:**

We designed a retrospective cohort study based on the Indonesian National Health Insurance (NHI) sample data from 2016–2020. We defined healthcare utilization as monthly outpatient and inpatient visits related to chronic diseases at the hospital and primary healthcare levels per 10,000 NHI members. We used interrupted time series analysis to evaluate how the healthcare utilization patterns had changed due to the COVID-19 pandemic.

**Results:**

Overall, hospital outpatient visits decreased by 39% when the pandemic occurred (95% Confidence Interval (CI): 0.48,0.76), inpatient visits by 28% (95% CI: 0.62,0.83), and primary healthcare visits by 34% (95% CI:0.55, 0.81). For patients with CVDs and multimorbidity, hospital outpatient and inpatient visit rates were reduced by 36% and 38%, respectively and primary healthcare visits by 32%. Some insignificant differences in the reduction of out-and inpatient visits were observed across diagnosis groups and regions.

**Conclusion:**

Healthcare utilization among patients with chronic diseases decreased significantly during COVID-19 and consistently across different chronic diseases and regions. To cope with the unmet needs of healthcare utilization in the context of the pandemic, the healthcare system needs to be strengthened to cater to the needs of the population-at-risk, especially for patients with CVDs and multimorbidity.

**Supplementary Information:**

The online version contains supplementary material available at 10.1186/s12889-023-17568-6.

## What is already known on this topic

• The COVID-19 pandemic has presented significant disruptions and challenges to healthcare systems worldwide.

• Limited research on the impact of the COVID-19 pandemic on healthcare utilization, specifically for patients with cardiovascular diseases (CVDs) and multimorbidity, is available.

## What this study adds

• During the COVID-19 pandemic, there was a substantial decrease in healthcare utilization among patients with chronic diseases, observed both in hospital and primary healthcare settings. This reduction was consistent across various diagnostic groups and regions in Indonesia.

• There were some insignificant differences in the reduction of healthcare utilization across groups of diagnoses, but some results point to a more significant reduction in relatively less developed regions.

## How this study might affect research, practice or policy

• Implementing an active non-communicable diseases screening program targeting vulnerable populations with chronic diseases to prevent premature mortality from non-communicable diseases during a pandemic, alongside efforts to strengthen the healthcare system and minimize the unmet healthcare needs.

## Background

The COVID-19 pandemic has posed significant disruptions and challenges to healthcare systems in many countries. Studies in different healthcare settings (including but not limited to the United States, Canada, Europe, India, and China) have demonstrated a decrease in hospital admission [1–5], and healthcare utilization for patients with chronic diagnoses such as stroke [6] during the COVID-19 pandemic. However, little is known about how the pandemic has affected healthcare utilization among patients with Cardiovascular diseases (CVDs) and those with multimorbidity.

CVDs were the leading cause of disability and death in Indonesia in 2019 [7]. Patients with CVDs require long-term continuous interactions with the health system to prevent complications and disability. Unmet healthcare needs among patients with CVDs could result in significant long-term clinical and economic burdens [1]. In addition, over one-third of Indonesian adults aged > 40 live with multimorbidity [8], a condition associated with higher healthcare utilization and spending [9–11]. Access to healthcare is affected by the availability of services and geographical location [12, 13], and healthcare is much less accessible in less developed and remote areas in an archipelagic nation such as Indonesia.

The National Health Insurance (NHI) Program in Indonesia, also known as *Jaminan Kesehatan Nasional* (*JKN)*, was launched as a mandatory program in 2014 to remove the economic barriers to access care for the Indonesian population. The NHI covered about 88% of the Indonesian population in 2022. The unreached 12% of the population are informal workers and those unwilling or unable to pay NHI premiums [14]. The NHI covers all medical costs for treatment in primary healthcare centers and hospitals without cost-sharing policies [15]. The NHI reimburses primary healthcare centers based on capitation and hospitals based on Diagnosis Related Groups. The NHI programme has contributed to increasing accessibility and utilization of outpatient and inpatient services in Indonesia since its conception [16, 17]. The NHI report indicates that the reimbursement for Noncommunicable diseases (NCDs) treatment was higher than for all other diagnoses between 2014 and 2019 [18] and is projected to increase in 2023–2026 [19].

In response to the COVID-19 pandemic, the Indonesian government enacted large-scale social restrictions by the end of March 2020 [20]. In April 2020, the government regulated how hospitals and primary healthcare centers should adapt and deliver healthcare services during the pandemic, with a restricted number of visits and provision of Personal Protective Equipment (PPE) for healthcare workers [21]. Although the restriction and COVID-19 response were nationally regulated, implementation and compliance varied across districts [22]. Consequently, the effects of COVID-19 on healthcare provision also varied. The impact of COVID-19 disruption on healthcare utilization among chronic disease patients in Indonesia is unknown.

This study analyzes the impacts of COVID-19 on healthcare utilization among adults aged 30 years and older under the NHI program, specifically those with CVDs and other chronic disease multimorbidity, across regions in Indonesia.

## Methods

### Study population

This retrospective cohort study was based on the NHI sample dataset, covering about 1% of all NHI members in Indonesia [23]. The sample dataset contained NHI membership and utilization data based on capitation-based primary care services, non-capitation-based primary care services, and hospital care services. The membership data contains sociodemographic information, the type of NHI membership, i.e., PBI [government-subsidized members], PPU [formal workers], BP [retired workers members], and PBPU [informal workers], and a sample weight variable. The healthcare utilization data consisted of variables on healthcare visits, ICD-10 codes diagnosis, procedure (ICD 9CM), the tariff of care, length of stay, referral care, and discharge status.

The NHI sample dataset included individuals and households registered up to 2020. The dataset was created based on a stratified random sample of 1% of the total 73,441,160 households enrolled at 22,024 primary healthcare centers across 514 districts in Indonesia. The NHI selected the sample from households registered at each of the primary healthcare centers, based on their healthcare utilization, i.e. (1) Households that never utilized healthcare services, (2) Households that have ever visited only primary healthcare centers, and (3) Households that have ever utilized primary healthcare center and hospital services. If the primary healthcare centers served all three types of households, then there were three strata in the primary healthcare centers. A total of 10 households were randomly sampled in each stratum, and all the individuals in the household were included.

The sample dataset consisted of 2,200,960 individuals who lived in 823,557 households. We excluded individuals aged below 30 (*n* = 989,484) and above 108 (*n* = 79), and those who passed away before 2016 (*n* = 36,551), resulting in a total of 1,210,924 eligible individuals. In this study, we included 378,495 unique individuals with chronic disease enrolled during the study period (Figure S1). The region is composed of five areas, each representing provinces that belong to a group of NHI tariff regulations regulated by the Ministry of Health (Figure S2).

### Measurements

The outcome was healthcare utilization for individuals with chronic disease. We defined healthcare utilization as monthly outpatient and inpatient visits related to chronic disease at the hospital and primary healthcare levels per 10,000 NHI members. We applied sample weights to calculate the monthly outpatient and inpatient rates.

CVDs and chronic diseases [24] were based on the list of ICD-10 codes represented in the Global Burden of Disease [25] (Table S1). Multimorbidity was defined as the presence of two or more chronic diseases [26] other than CVDs. We stratified NHI members into five groups based on the presence of CVDs and/or multimorbidity of chronic diseases: (1) NHI members with no CVDs but with single chronic morbidity, (2) NHI members with no CVDs but with multimorbidity, (3) NHI members with CVDs diagnoses but no comorbidity, (4) NHI members with CVDs and one comorbidity, and (5) NHI members with CVDs and multimorbidity.

### Statistical analyses

In the descriptive analysis (Table 1), we compared the relative changes in the average healthcare utilization rates before (January 2016-March 2020) and during the COVID-19 pandemic (March 2020-December 2020). We assessed the relative change across the five disease groups and regions.

**Table 1:** Relative change in the monthly rate of healthcare utilization related to chronic diseases before and during the COVID-19 pandemic

Outcome	Utilization rate before the COVID-19 pandemic Mean (SD)			Utilization rate during COVID-19 pandemic Mean (SD)			Relative Change in utilization rates before and during COVID-19 pandemic^e^		
	**Hospital Outpatient**	**Hospital Inpatient**	**PHC Outpatient**	**Hospital Outpatient**	**Hospital Inpatient**	**PHC Outpatient**	**Hospital Outpatient**	**Hospital Inpatient**	**PHC Outpatient**
**Overall rate at national level**^**a**^	286.90 (67.43)	31.47 (3.36)	499.99 (15.95)	243.77 (38.56)	20.56 (4.05)	539.35 (75.68)	-15.03%	-34.67%	7.8%
**Overall rate across different Regions**^**b**^									
Region 1	338.50 (78.68)	31.96 (3.56)	562.36 (174.43)	287.09 (43.15)	21.39 (4.65)	604.12 (85.22)	-15.19%	-33.07%	7.43%
Region 2	287.07 (64.62)	28.57 (4.13)	504.50 (153.15)	238.16 (40.60)	19.40 (3.42)	536.22 (74.00)	-17.04%	-32.10%	6.29%
Region 3	195.11 (54.25)	34.81 (4.13)	430.63 (155.79)	174.13 (33.99)	21.11 (4.14)	477.68 (66.45)	-10.75%	-39.36%	10.93%
Region 4	240.52 (60.80)	32.37 (4.96)	382.76 (137.97)	202.98 (36.08)	19.32 (4.22)	401.09 (62.94)	-15.61%	-40.32%	4.79%
Region 5	94.01 (15.37)	19.55 (3.13)	140.86 (46.54)	76.05 (12.75)	13.21 (3.20)	172.89 (26.68)	-19.10%	-32.43%	22.74%
**Rates among chronic disease patients across diagnosis groups**^**c**^									
(1) No CVDs, but with single chronic morbidity	102.22 (24.51)	12.28 (1.27)	314.53 (98.86)	76.83 (15.42)	7.89 (1.51)	325.75 (51.15)	-24.84%	-35.75%	3.57%
(2) No CVDs, but with multimorbidity	61.94 (10.88)	7.62 (0.81)	89.56 (20.02)	47.70 (7.23)	4.73 (0.88)	84.65 (10.51)	-22.99%	-37.93%	-5.48%
(3) CVDs, but no comorbidity	21.69 (5.29)	2.63 (0.40)	23.69 (11.11)	16.39 (2.81)	1.54 (0.32)	30.73 (4.00)	-24.44%	-41.44%	29.72%
(4) CVDs and one comorbidity	37.93 (12.48)	3.56 (0.61)	32.33 (14.77)	38.54 (5.76)	2.58 (0.76)	44.64 (5.19)	1.61%	-27.53%	38.08%
(5) CVDs and multimorbidity	63.10 (16.84)	5.36 (1.04)	39.87 (16.34)	64.28 (8.03)	3.79 (0.76)	53.56 (5.61)	1.87%	-29.29%	34.34%
**Rates among patients with CVDs and multimorbidity across regions**^**d**^									
Region 1	77.72 (18.87)	5.88 (1.08)	44.87 (18.06)	77.70 (9.40)	3.97 (0.81)	60.94 (6.10)	-0.03%	-32.48%	35.81%
Region 2	59.55 (18.11)	4.57 (1.37)	43.58 (17.93)	60.73 (8.09)	3.86 (1.15)	57.27 (6.86)	1.98%	-15.54%	31.41%
Region 3	36.75 (14.78)	4.95 (1.39)	32.36 (14.03)	44.25 (6.06)	3.77 (0.88)	43.61 (5.65)	20.41%	-23.84%	34.77%
Region 4	51.77 (17.49)	5.45 (2.09)	31.09 (15.27)	43.93 (7.46)	3.33 (0.75)	35.35 (3.37)	-15.14%	-38.90%	13.70%
Region 5	17.41 (6.63)	2.85 (1.28)	9.71 (5.02)	17.49 (3.60)	2.09 (1.05)	14.57 (2.12)	0.46%	-26.67%	50.05%

The monthly rate was presented per 10,000 NHI members

^a^Numerator was the number of visits with chronic diseases (overall group). The denominator was the number of NHI members at the national level. PHC = Primary Healthcare

^b^Numerator was the number of visits with chronic diseases (overall group). The denominator was the number of NHI members at the regional level

^c^Numerator was the number of visits with chronic diseases in each group. The denominator was the number of NHI members at the national level

^d^The numerator was the number of visits by NHI members with CVDs and multimorbidity per region, and the denominator was the total NHI member population per region. See Supplementary Figure S2 for details about the regions

^e^ $$\mathrm{Relative Change}=\frac{\mathrm{visit rates during COVID}-19-\mathrm{visit rates before COVID}-19}{\mathrm{visit rates before COVID}-19}$$

We used the interrupted time series (ITS) analysis to analyze the healthcare utilization patterns before and during the COVID-19 pandemic. We modeled healthcare utilization using Poisson regression, as the healthcare utilization data was assumed to follow the Poisson distribution with scaling adjustment to control for potential over-dispersion [27].

$${{{\text{Y}}}_{t}=\upbeta }_{0}+{\upbeta }_{1}T+{\upbeta }_{2}{X}_{t}+{\beta }_{3 }\left(T-{T}_{0}\right){X}_{t} +{e}_{t}$$

Y_t_ represents the outcome variable at time t, T is the time elapsed in months from January 2016, X_t_ is a dummy variable capturing the pre-COVID-19 period (coded 0) and the COVID-19 period (coded 1). ${\upbeta }_{0}$ represents the intercept of the model, ${\upbeta }_{1}$ captures the time trend of the outcome variable underlying pre-COVID trend, ${\upbeta }_{2}$ captures the immediate level change in the outcome variable when entering the COVID-19 period, and ${\upbeta }_{3}$ indicates the change in the trajectory (slope) of the outcome variable during the COVID-19 period (${T}_{0}$ as the beginning of the COVID-19). The error term (${e}_{t}$) at time t represents the random variability not explained by the model. The Incidence Rate Ratio (IRR) was obtained by exponentiating the Poisson regression coefficient (${\upbeta }_{2}),$ which provided the change in monthly levels of visits due to entering the Covid-19 period [28].

We also performed the ITS analysis stratified by the five diagnosis groups to examine the heterogeneous effects of COVID-19 disruptions experienced by patients with different diagnoses. For the overall and diagnosis-stratified analyses, we utilized NHI members at the national level as the denominator in calculating the outcome rates. For patients with CVDs and multimorbidity, we stratified the analysis by geographical regions. For this analysis, we used NHI members in each region as the denominator in calculating the outcome variables. All analyses were performed, controlling for the seasonal pattern by additive Fourier Poisson time-series regression models, and were carried out using Bernal’s procedures [27].

We conducted statistical significance tests (assuming independence between groups) to assess if the impact of the COVID-19 period on utilization differed across diagnosis groups and regions. Group (5) CVDs and multimorbidity and Region 1 were used as the reference group.

The analyses were performed using Stata/SE 17 (Stata Corp LLC, 2021).

### Ethics approval and consent to participate

This study was approved by the Research Ethics Committee of Atma Jaya Catholic University of Indonesia (Number:0002S/III/PPPE.PM.1005/03/2023), which also waived the need for informed consent. Written informed consent for participation was not required for the collection and utilisation of administrative health data in accordance with national legislation and institutional requirements. All research steps were carried out in accordance with relevant guidelines and regulations.

## Results

### Descriptive analysis of healthcare utilization before and during COVID-19

The national average monthly outpatient and inpatient hospital visit rate was lower during the COVID-19 pandemic compared to the period before the pandemic (Table 1). The average visit rates were 34.67% lower for inpatient care and 15.03% lower for outpatient care during the pandemic. The decline in average monthly rates of hospital visits varied across different regions, with a range of 32.10% to 40.32% lower for inpatient visits and 10.75% to 19.10% lower for outpatient visits.

For patients in different chronic disease diagnosis groups, we observed a consistent decrease in inpatient hospital visit rates (Table 1). We also observed reductions in outpatient hospital visit rates for patients with No CVDs but with single chronic morbidity, No CVDs but with multimorbidity, and CVDs but no comorbidity. In contrast, the primary healthcare center chronic disease visits increased across all diagnosis groups except for patients with No CVDs but with multimorbidity.

For CVDs and multimorbidity patients, we observed a 29.29% lower inpatient visit rate, but a higher outpatient visit rate of 1.87% at hospitals and 34.34% at primary healthcare during the pandemic. The inpatient visit rate reduction was consistent across regions, ranging from -15.54% to -38.90%. The outpatient visit rate increment was also consistent across regions, ranging from 13.70% to 50.05% for outpatient visits rate at primary healthcare.

### Effects of COVID-19 disruptions on healthcare utilization

**Fig. 1:**
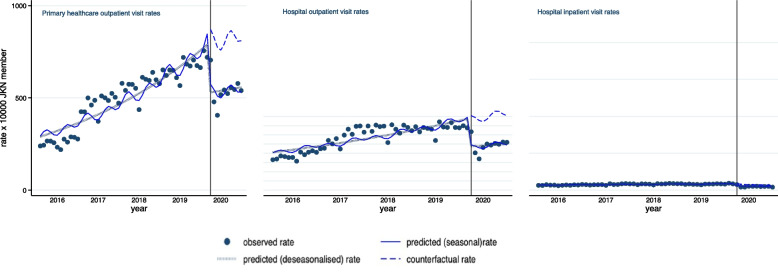
Monthly hospital and primary healthcare visit rates before and during COVID-19 at the national level

The ITS analysis showed a significant reduction in the monthly out- and inpatient visits rate for patients with chronic diseases during COVID-19 (Fig. 1, Figure S3). We would have expected the hospital outpatient visit rate to be more than 400 per 10,000 JKN members per month had the COVID-19 disruption not occurred, but during COVID-19, the outpatient visit rate was 279.85 per 10,000 (Fig. 1). During the COVID-19 period, average hospital outpatient visits decreased by 39% (Incidence Rate Ratio (IRR):0.61; 95% Confidence Interval (CI): 0.48, 0.76), by 28% for inpatient visits (IRR 0.72; 95% CI: 0.62, 0.83), and by 34% for primary healthcare center (PHC) (IRR 0.66; 95% CI:0.55, 0.81) (Table 2).

**Table 2:** Effect of COVID-19 disruptions on monthly hospital and PHC visit rates in Indonesia

Outcome	Hospital		PHC
	**Outpatient visits** **IRR (CI)**	**Inpatient visits** **IRR (CI)**	**Outpatient visits** **IRR (CI)**
**Overall healthcare utilization related to chronic diseases at national level**^**a**^	0.61 (0.48,0.76)	0.72 (0.62,0.83)	0.66 (0.55,0.81)
**Healthcare utilization among chronic disease patients in different diagnosis groups**^**b**^			
(1) No CVDs, but with single chronic morbidity	0.59 (0.44,0.79)	0.75 (0.64,0.87)	0.66 (0.53,0.82)
(2) No CVDs, but with multimorbidity	0.58 (0.48,0.72)	0.71 (0.60,0.84)	0.69 (0.59,0.80)
(3) CVDs, but no comorbidity	0.57 (0.44,0.74)	0.77 (0.60,0.99)	0.62 (0.49,0.78)
(4) CVDs and one comorbidity	0.62 (0.49,0.79)	0.76 (0.60,0.96)	0.63 (0.52,0.76)
(5) CVDs and multimorbidity	0.64 (0.54,0.75)	0.62 (0.51,0.75)	0.68 (0.57,0.80)
**Healthcare utilization among patients with CVDs and multimorbidity across different regions**^**c**^			
Region 1	0.65 (0.55,0.78)	0.64 (0.51,0.79)	0.69 (0.59,0.82)
Region 2	0.59 (0.51,0.69)	0.66 (0.47,0.93)	0.67 (0.58,0.77)
Region 3	0.62 (0.48,0.79)	0.57 (0.43,0.76)	0.66 (0.52,0.83)
Region 4	0.47 (0.35,0.64)	0.48 (0.29,0.77)	0.50 (0.39,0.65)
Region 5	0.71 (0.49,1.01)	0.53 (0.26,1.08)	0.61 (0.47,0.80)

The effect of COVID-19 disruption on monthly hospital and PHC visit rates was presented among patients in different diagnosis groups and among patients with CVDs and multimorbidity across different regions in Indonesia

The Incidence Rate Ratio (IRR) was obtained by exponentiating the Poisson regression coefficient (β_2_) which provided the change in monthly levels of visits due to entering the Covid-19 period. PHC Primary Healthcare

^a^The IRR for patients with chronic diseases at the national level

^b^The IRR for patients in different diagnosis groups at the national level

^c^The IRR for patients with CVDs and multimorbidity at the regional level. See Supplementary Figure S2 for details about the regions

### Heterogeneity of effects in COVID-19 disruptions among patients with different diagnoses

In general, the reductions in visit rate were similar across patients with different diagnoses, and only a few differences between the groups (Supplementary Table S2).

**Fig. 2:**
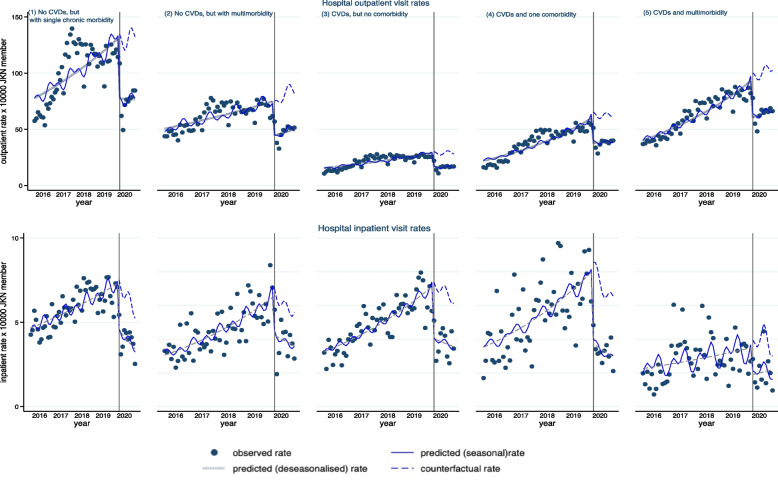
Monthly hospital outpatient and inpatient visit rates before and during COVID-19 across groups of diagnosis

Patients with CVDs and multimorbidity experienced a 36% reduction in outpatient visit rate (IRR 0.64; 95% CI:0.54, 0.75), while patients with CVDs, but no comorbidity experienced a 43% reduction (IRR 0.57; 95% CI:0.44, 0.74) (Table 2). We observed an increasing trend in the outpatient rate before and during COVID-19 (Fig. 2). Patients with no CVDs but with multimorbidity had a 42% reduction (IRR 0.58; 95% CI: 0.48, 0.72) in outpatient visit rate during COVID-19. Patients with no CVDs but with single chronic morbidity experienced a 41% reduction in outpatient visit rate (IRR 0.59; 95% CI:0.44, 0.79) due to the pandemic. The differences in the decrease in hospital outpatient visit rates were not statistically significant across the group of diagnoses (Supplementary Table S2).

The reduction in primary healthcare visit rates ranges from 31% among patients with no CVDs but with multimorbidity (IRR 0.69; 95% CI:0.59, 0.80) to 38% in patients with CVDs but no comorbidity (IRR 0.62; 95% CI:0.49, 0.78) (Table 2). The reductions in primary healthcare visit rates across a group of diagnoses were relatively similar (Supplementary Table S2).

The reduction in inpatient visit rates ranged from 24% among patients with CVDs, and one comorbidity (IRR:0.76; 95% CI:0.60, 0.96) to 38% among patients with CVDs and multimorbidity (IRR:0.62; 95% CI:0.51, 0.75) (Table 2). A lower reduction in inpatient visit rates was observed among patients with CVDs but no comorbidity compared to patients with CVDs and multimorbidity (mean difference:-0.07; 95% CI:-0.25, 0.11). However, the reductions in inpatient visit rates across other diagnoses were relatively similar compared to patients with CVDs and multimorbidity (Supplementary Table S2). In addition, we also observed a significant slope change in the overall inpatient visit rates during COVID-19, which showed a declining trend compared to the increasing slope trend before COVID-19 (Fig. 2).

### Heterogeneity of effects of COVID-19 among patients with CVDs and multimorbidity across regions

**Fig. 3:**
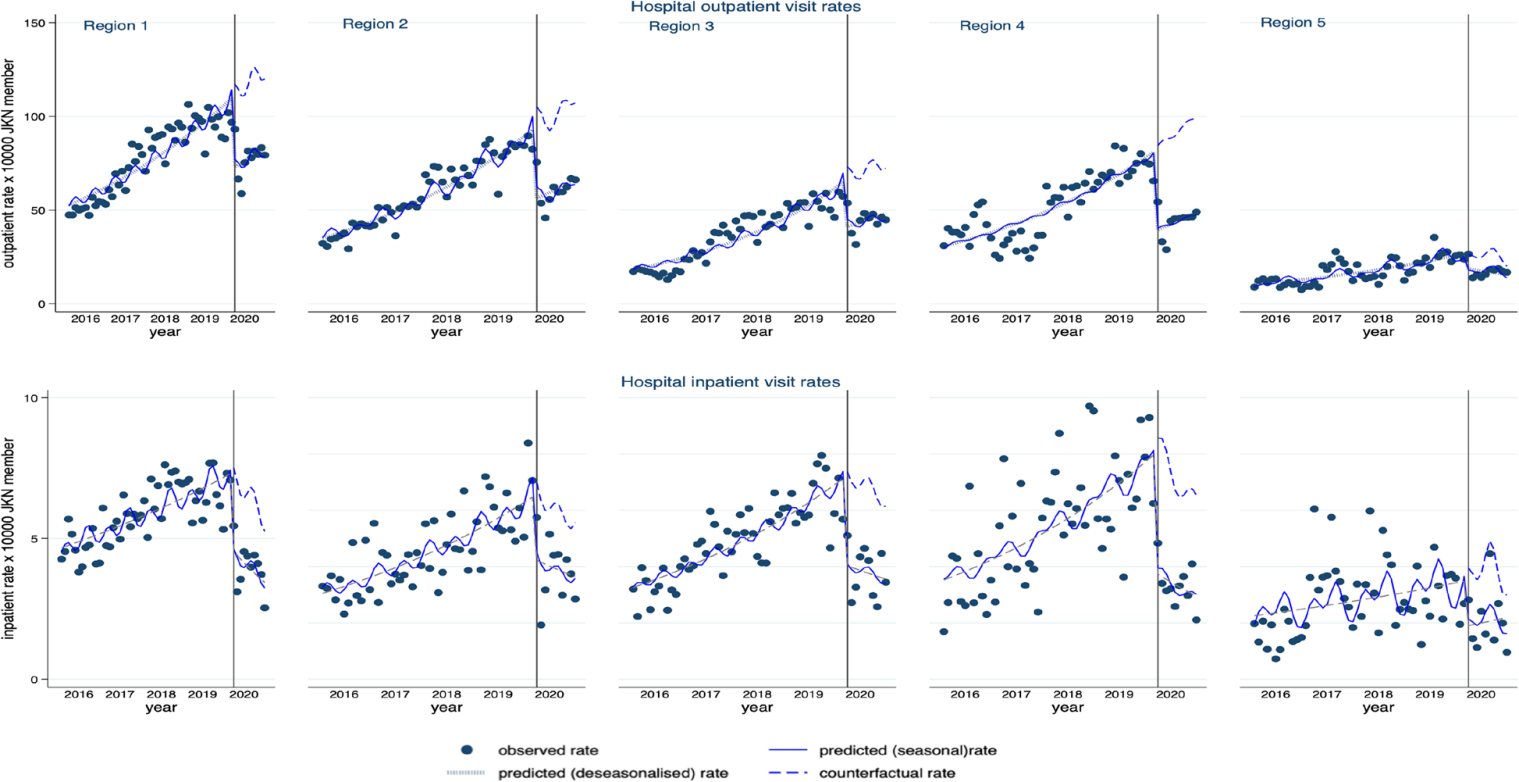
Monthly hospital outpatient and inpatient visit rates among patients with CVDs and multimorbidity across regions

We observed differences in the reduction of out- and inpatient visit rates among patients with CVDs and multimorbidity across regions (Fig. 3). The reduction in out- and inpatient visit rates ranged from 38% in Region 3 to 53% in Region 4 for outpatient rates and from 34% in Region 2 to 52% in Region 4 for inpatient visit rates (Table 2). We observed a difference in the reduction of outpatient visit rates in Region 4 compared to Region 1 (mean difference: -0.19, 95%CI: -0.36, 0.22), but insignificant. A relatively similar reduction across regions was also observed for inpatient visit rates and outpatient visit rates at primary healthcare (Supplementary Table S2).

## Discussion

Our analysis indicates substantial reductions in healthcare utilization at the hospital and primary healthcare centers by patients with chronic disease in Indonesia during the COVID-19 pandemic. These reductions were observed consistently across diagnosis groups and regions in Indonesia.

The COVID-19 pandemic has posed significant challenges for patients to access healthcare due to reduced supply and demand. On the supply side, strict health protocols were implemented at healthcare facilities to minimize the spread of COVID-19, with a restricted number of patients allowed to access care at the hospitals, and mandatory protection measurement by providing healthcare workers with PPE. Healthcare facilities that failed to equip their staff with PPE to meet the standard health protocols had to forgo service to the patients [29]. To flatten the pandemic curve and preserve healthcare capacity during the pandemic, healthcare facilities delayed the management of elective cases and patients with non-COVID-19 diagnoses to focus on providing care to COVID patients with severe conditions needing intensive care [30].

On the demand side, the reduction of healthcare utilization was driven by patients' decision to delay care-seeking due to the risk and fear of COVID-19 infection [4, 29]. Healthcare utilization at hospitals and primary healthcare also decreased due to the social restriction policy, which paved the way for the expansion of telemedicine use in the country. Telemedicine-based services were piloted in September 2020, allowing consultations for patients with chronic diseases at the primary healthcare level and reducing the need for in-person care at the hospital [31]. As the use of telemedicine was not part of the current NHI coverage, we could not quantify the extent to which the use of telemedicine has buffered the effects of foregone healthcare utilization during the COVID-19 pandemic.

The effects of the COVID-19 pandemic on healthcare utilization are relatively homogenous among patients with different chronic diseases. The pandemic could affect patients differently depending on the disease’s complexity. The pandemic affected patients with less severe diseases, particularly those with averted outpatient visits as reported in South Korea [32], and eight European countries [33]. Reductions in in-person healthcare utilization were also observed among Chronic Kidney Disease (CKD) patients enrolled in commercial and Medicare Advantage health plans in the USA [34]. Notably, those with stage G4 CKD and a younger age exhibited higher odds of experiencing a greater healthcare deficit [34]

There were some differences in the effects of COVID-19 on the reduction of healthcare utilization among patients with CVDs and multimorbidity across different regions in Indonesia. The observed reduction was less among patients in Region 1 on Java Island, where 56.2% of the population lives [35], 50.5% of the healthcare resources are spent [36], and 50% of referral hospitals are located [37], consequently with better access to healthcare services [18]. This unequal pattern of healthcare utilization, with higher utilization in better-resourced regions, was also observed before the pandemic [13, 18]. With better resources, the healthcare system in Region 1 was more resilient in coping with the pandemic, ensuring a sufficient supply of healthcare services [22].

The reduction in healthcare utilization among patients with CVDs during the pandemic has detrimental consequences for those needing continuous treatment. CVDs have consistently been ranked as a leading cause of death in Indonesia [7]. Prior to the pandemic, nearly 70% of patients at risk for CVDs failed to receive appropriate CVDs treatments [38]. Non-compliance with treatments for individuals with CVDs increases their long-term health risks, thereby affecting their sustained care requirements and amplifying the economic burdens for the patients, their families and society [39].

## Strengths and limitations

This study used a quasi-experimental design to analyze the impact of the COVID-19 pandemic on healthcare utilization using a large national administrative insurance database. Moreover, this study investigated the impact of COVID-19 across different combinations of chronic diagnoses and CVDs and portrayed the heterogeneity of COVID-19 disruption effects across regions.

The quality of this national health insurance administrative data depends on the coding system used across different healthcare facilities. The validity and reliability of the data, which has not been established in any study, could potentially influence the quality of the estimate of disease burdens reported in this study. In addition, we cannot compare healthcare utilization among those with unmet needs as information about needs and if needs were met do not exist in the NHI database. Moreover, despite the importance of understanding the impacts of foregone healthcare utilization, particularly among individuals with CVDs multimorbidity on subsequent mortality, the National Health Insurance database is not readily linked with the death register, rendering investigation on this issue not yet possible.

The costs for COVID-19 patients treated in hospitals were reimbursed directly by the Ministry of Health and not through the National Health Insurance. Consequently, the NHI database does not fully capture the healthcare utilization of COVID-19 patients [40], and therefore, estimates of healthcare utilization rates during COVID-19 based on the NHI database might be underestimated.

## Policy implications

Evidence from this study calls for attention to support the vulnerable population, especially patients with chronic disease during the pandemic. Many patients with chronic disease have been affected by deferred care and foregone health services that could lead to increased disability and premature mortality [1, 41]. The pandemic control policies (including providing COVID-19 vaccination to the population and providing protective devices for healthcare staff) contribute to the recovery of healthcare utilization. This recovery is achieved through the improvement of both the supply and demand of healthcare services [42].

Further studies are needed to explore the effect of regional policy intervention, healthcare readiness, individual factors, unmet needs and social determinants of health to explain the change in health-seeking behavior and healthcare utilization in the population during the pandemic.

## Conclusion

In Indonesia, healthcare utilization among patients with chronic disease under NHI dropped significantly during the COVID-19 pandemic. The reduction was observed in out- and inpatient hospital and primary healthcare center visits. The averted healthcare utilization was different across chronic diseases and regions but insignificant. Policy intervention is essential to ensure the availability and accessibility of care and health services in the context of the pandemic. It is important to implement active NCDs screening and control programs to reduce the subsequent health and economic burden related to NCDs due to delayed treatment during the pandemic.

### Supplementary Information

**Additional file 1:** **Figure S1.** Hierarchy of NHI data structure 2016-2020: inclusion and exclusion criteria. **Figure S2.** Indonesian map with 5 regions based on the national health insurance tariff group. **Table S1.** List of ICD-10 codes for noncommunicable diseases. **Figure S3.** Monthly hospital inpatient rates before and during COVID-19 at the national level (2016-2020). **Table S2.** Difference in the mean estimate of COVID-19’s effect (IRR) on monthly visit rates by a group of diagnosis and patients with CVDs and multimorbidity by regions. **Figure S4.** Monthly primary healthcare outpatient visit rates before and during COVID-19 across groups of diagnosis at the national level (2016-2020). *Interrupted Time Series Analysis in five diagnosis groups. **Figure S5.** Monthly primary healthcare outpatient visit rates among patients with CVDs and multimorbidity before and during COVID-19 across regions in Indonesia. Interrupted time series analysis stratified by five regions in Indonesia. The regions are listed under supplementary Figure S2.

## Data Availability

The data from National Social Security Agency for Health that support the findings of the study were used under license for the current study, and so are not publicly available. Data are however available from the authors upon reasonable request and with permission of National Social Security Agency for Health. The data were de-identified, and researchers interested in using this dataset can find details at https://bpjs-kesehatan.go.id/#/

## References

[1] World Health Organization. The impact of the COVID-19 pandemic on noncommunicable disease resources and services: results of a rapid assessment. 2020. Accessed.

[2] Birkmeyer JD, Barnato A, Birkmeyer N, Bessler R, Skinner J (2020). The impact of the COVID-19 pandemic on hospital admissions in the United States: study examines trends in US hospital admissions during the COVID-19 pandemic. Health Aff.

[3] Rennert-May E, Leal J, Thanh NX, Lang E, Dowling S, Manns B (2021). The impact of COVID-19 on hospital admissions and emergency department visits: A population-based study. PLoS One.

[4] Xiao H, Dai X, Wagenaar BH, Liu F, Augusto O, Guo Y (2021). The impact of the COVID-19 pandemic on health services utilization in China: Time-series analyses for 2016–2020. The Lancet Regional Health-Western Pacific.

[5] Roy CM, Bollman EB, Carson LM, Northrop AJ, Jackson EF, Moresky RT (2021). Assessing the indirect effects of COVID-19 on healthcare delivery, utilization and health outcomes: a scoping review. Eur J Public Health.

[6] Desai SM, Guyette FX, Martin-Gill C, Jadhav AP (2020). Collateral damage - Impact of a pandemic on stroke emergency services. J Stroke Cerebrovasc Dis.

[7] Institute for Health Metrics and Evaluation .Global Burden of Diseases: Indonesia. 2019. Available: https://www.healthdata.org/data-visualization/gbd-compare. Accessed.

[8] Hussain MA, Huxley RR, Al MA (2015). Multimorbidity prevalence and pattern in indonesian adults: an exploratory study using national survey data. BMJ Open.

[9] Madyaningrum E, Chuang Y-C, Chuang K-Y (2018). Factors associated with the use of outpatient services among the elderly in Indonesia. BMC Health Serv Res.

[10] Marthias T, Anindya K, Ng N, McPake B, Atun R, Arfyanto H (2021). Impact of non-communicable disease multimorbidity on health service use, catastrophic health expenditure and productivity loss in Indonesia: a population-based panel data analysis study. BMJ Open.

[11] Anindya K, Ng N, Atun R, Marthias T, Zhao Y, McPake B (2021). Effect of multimorbidity on utilisation and out-of-pocket expenditure in Indonesia: quantile regression analysis. BMC Health Serv Res.

[12] Sambodo NP, Van Doorslaer E, Pradhan M, Sparrow R (2021). Does geographic spending variation exacerbate healthcare benefit inequality? A benefit incidence analysis for Indonesia. Health Policy Plan.

[13] Mulyanto J, Kunst AE, Kringos DS. The contribution of service density and proximity to geographical inequalities in health care utilisation in Indonesia: A nation-wide multilevel analysis. Journal of Global Health. 2020;10. 10.7189/jogh.10.020428 PMC7719271 33312501

[14] National Health Insurance Agency. Number of JKN Member (In Indonesian: BPJS Kesehatan. Peserta Program JKN). 2022. Available: https://faskes.bpjs-kesehatan.go.id/aplicares/#/app/peta. Accessed.

[15] Agustina R, Dartanto T, Sitompul R, Susiloretni KA, Achadi EL, Taher A, et al. Universal health coverage in Indonesia: concept, progress, and challenges. The Lancet. 2018. 10.1016/S0140-6736(18)31647-7 30579611

[16] Erlangga D, Ali S (2019). Bloor KJIjoph. The impact of public health insurance on healthcare utilisation in Indonesia: evidence from panel data.

[17] Johar M, Soewondo P, Pujisubekti R, Satrio HK, Adji A (2018). Inequality in access to health care, health insurance and the role of supply factors. Soc Sci Med.

[18] DJSN and BPJS Kesehatan. JKN Statistic 2015–2019 (In Indonesian: DJSN dan BPJS Kesehatan. Statistik JKN 2015–2019). Jakarta2021 [cited 2021 13 July 2021].

[19] BPJS Kesehatan. Documentation of Baseline Projection of Social Security Fund for the Period 2022–2026 (In Indonesian: BPJS Kesehatan. Dokumentasi Proyeksi Baseline Dana Jaminan Sosial Periode 2022–2026). 2022.

[20] Government Regulation. Government Regulation on Large-Scale Social Restrictions in the Context of Accelerating the Handling of Corona Virus Disease 2019 (COVID-19) 2020 (In Indonesian: Peraturan Pemerintah.Peraturan Pemerintah (PP) tentang Pembatasan Sosial Berskala Besar dalam Rangka Percepatan Penanganan Corona Virus Disease 2019 (COVID-19)). 2020. Available: https://peraturan.bpk.go.id/Home/Details/135059/pp-no-21-tahun-2020. Accessed.

[21] Circular Letter Number: HK.02.02/11/509/2020 Regarding Family Health Services in the Era of the COVID-19 Pandemic (In Indonesian: Kementerian Kesehatan. Surat Edaran Nomor: HK.02.02/11/509/2020 Tentang Pelayanan Kesehatan Keluarga di Era Pandemi COVID-19), (2020).

[22] TNP2K. Assessment of The Potential Effects of Policy Responses and Public Financial Management (PFM) Adjustment for the Continuation of Four Essential Public Health Services at the Primary Health Care Centre (PHC) Level During the COVID-19 Pandemic in Indonesia. Jakarta, Indonesia: TNP2K: 2021.

[23] Ariawan I, Sartono B, Jaya C, Mawardi J, Sodiq J, Baros W, et al. BPJS Health Sample Data for 2015–2021 (In Indonesian: Data Sampel BPJS Kesehatan Tahun 2015–2021). Jakarta: BPJS Kesehatan. 2022.

[24] Agency for Healthcare Research and Quality R, MD,. Tools Archive for the Chronic Condition Indicators for ICD-10-CM. Healthcare Cost and Utilization Project (HCUP). 2020. Available: https://www.hcup-us.ahrq.gov/toolssoftware/chronic_icd10/chronic_icd10.jsp#overview. Accessed.

[25] Institute for Health Metrics and Evaluation. Global Burden of Disease Study 2017 (GBD 2017) Causes of Death and Nonfatal Causes Mapped to ICD Codes. 2017. Available: http://ghdx.healthdata.org/record/ihme-data/gbd-2017-cause-icd-code-mappings. Accessed.

[26] Koller D, Schön G, Schäfer I, Glaeske G, van den Bussche H, Hansen H (2014). Multimorbidity and Long-term Care Dependency—a five-year follow-up. BMC Geriatr.

[27] Bernal JL, Cummins S, Gasparrini A (2017). Interrupted time series regression for the evaluation of public health interventions: a tutorial. Int J Epidemiol.

[28] Bernal JL, Cummins S, Gasparrini A (2021). Corrigendum to: Interrupted time series regression for the evaluation of public health interventions: a tutorial. Int J Epidemiol.

[29] Save The Children and Center for Health Economics and Policy Studies. Health Service Access for Children in Bandung and West Sumba Districts. 2020.

[30] Coordinating Ministry for Economic Affairs of the Republic of Indonesia and the Committee for COVID-19 Handling and National Economic Recovery. COVID-19 vaccination in Indonesia (In Indonesian: Kementerian Koordinator Bidang Perekonomian Republik Indonesia dan Komite Penanganan COVID-19 dan Pemulihan Ekonomi Nasional. Vaksinasi COVID-19 di Indonesia). 2022.

[31] Stamenova V, Chu C, Pang A, Fang J, Shakeri A, Cram P (2022). Virtual care use during the COVID-19 pandemic and its impact on healthcare utilization in patients with chronic disease: A population-based repeated cross-sectional study. PLoS One.

[32] Kang E, Yun J, Hwang SH, Lee H, Lee JY (2022). The impact of the COVID-19 pandemic in the healthcare utilization in Korea: Analysis of a nationwide survey. J Infect Public Health.

[33] Selke Krulichová I, Selke GW, Bennie M, Hajiebrahimi M, Nyberg F, Fürst J (2022). Comparison of drug prescribing before and during the COVID-19 pandemic: A cross-national European study. Pharmacoepidemiol Drug Saf.

[34] Diamantidis CJ, Cook DJ, Dunning S, Redelosa CK, Bartolome MFD, Romero RAA (2022). Missing Care: the Initial Impact of the COVID-19 Pandemic on CKD Care Delivery. J Gen Intern Med.

[35] Central Buerau Statistics of Indonesia Population Projection by Province and Sex (Thousand), 2018–2020 2020 (In Indonesian: BPS. Jumlah Penduduk Hasil Proyeksi Menurut Provinsi dan Jenis Kelamin (Ribu Jiwa), 2018–2020). 2020. Available: https://www.bps.go.id/indicator/12/1886/1/jumlah-penduduk-hasil-proyeksi-menurut-provinsi-dan-jenis-kelamin.html. Accessed.

[36] Ministry of Finance. Annual Data on the Realization of Health Expenditure at the Regency / City level throughout Indonesia for a period of 10 years, namely 2010 to 2019 (In Indonesian: Kementerian Keuangan. Data Tahunan Realisasi Belanja Kesehatan tingkat Kabupaten/Kota di seluruh Indonesia pada periode waktu 10 tahun, yaitu Tahun 2010 sampai dengan Tahun 2019). 2020. Available: https://djpk.kemenkeu.go.id/?p=20826. Accessed.

[37] Central Buerau Statistics of Indonesia. Number of General Hospitals, Special Hospitals, Puskesmas, Primary Clinics, and Posyandu by Province, 2021 (In Indonesian: BPS. Jumlah Rumah Sakit Umum, Rumah Sakit Khusus, Puskesmas, Klinik Pratama, dan Posyandu Menurut Provinsi, 2021). 2021. Available: https://www.bps.go.id/indikator/indikator/view_data_pub/0000/api_pub/biszcFRCUnVKUXNnTDZvWnA3ZWtyUT09/da_04/1. Accessed.

[38] Maharani A, Tampubolon G (2014). Unmet needs for cardiovascular care in Indonesia. PLoS ONE.

[39] Dunbar SB, Khavjou OA, Bakas T, Hunt G, Kirch RA, Leib AR (2018). Projected costs of informal caregiving for cardiovascular disease: 2015 to 2035: a policy statement from the American Heart Association. Circulation.

[40] Decree of the Minister of Health of the Republic of Indonesia No HK.01.07/Menkes/446/2020 concerning Technical Guidelines for Reimbursement Claims for Patient Services for Certain Emerging Infectious Diseases for Hospitals Organizing Corona Virus Disease 2019 (COVID-19) Services (In Indonesia: Keputusan Menteri Kesehatan Republik Indonesia No HK.01.07/Menkes/446/2020 tentang Petunjuk Teknis Klaim Penggantian Biaya Pelayanan Pasien Penyakit Infeksi Emerging Tertentu bagi Rumah Sakit yang Menyelenggarakan Pelayanan Corona Virus Disease 2019 (COVID-19), (2020).

[41] Ugarte MP, Achilleos S, Quattrocchi A, Gabel J, Kolokotroni O, Constantinou C (2022). Premature mortality attributable to COVID-19: potential years of life lost in 17 countries around the world, January–August 2020. BMC Public Health.

[42] Doubova SV, Arsenault C, Contreras-Sánchez SE, Borrayo-Sánchez G, Leslie HH. The road to recovery: an interrupted time series analysis of policy intervention to restore essential health services in Mexico during the COVID-19 pandemic. Journal of global health. 2022;12. 10.7189/jogh.12.05033 PMC9304921 35866236

