# Supplementary material for: Effects of the COVID-19 pandemic on healthcare utilization among older adults with cardiovascular diseases and multimorbidity in Indonesia: an interrupted time-series analysis
Source: BMC Public Health. 2024 Jan 2;24:71. doi: 10.1186/s12889-023-17568-6 (PMC10763491; doi:10.1186/s12889-023-17568-6)
Supplement: Supplementary file 1 — Additional file 1: Figure S1. Hierarchy of NHI data structure 2016-2020: inclusion and exclusion criteria. Figure S2. Indonesian map with 5 regions based on the national health insurance tariff group. Table S1. List of ICD-10 codes for noncommunicable diseases. Figure S3. Monthly hospital inpatient rates before and during COVID-19 at the national level (2016-2020). Table S2. Difference in the mean estimate of COVID-19’s effect (IRR) on monthly visit rates by a group of diagnosis and patients with CVDs and multimorbidity by regions. Figure S4. Monthly primary healthcare outpatient visit rates before and during COVID-19 across groups of diagnosis at the national level (2016-2020). *Interrupted Time Series Analysis in five diagnosis groups. Figure S5. Monthly primary healthcare outpatient visit rates among patients with CVDs and multimorbidity before and during COVID-19 across regions in Indonesia. Interrupted time series analysis stratified by five regions in Indonesia. The regions are listed under supplementary Figure S2. [file 12889_2023_17568_MOESM1_ESM.docx]

**Online Supplementary Materials**

Figure S1 Hierarchy of NHI data structure 2016-2020: inclusion and exclusion criteria

**Regions**

Region consists of five regions represent the following provinces: Banten, DKI Jakarta, West Java, Central Java, Yogyakarta and East Java (Region 1), West Sumatera, Riau, South Sumatera, Lampung, Bali and West Nusa Tenggara (Region 2); Aceh, North Sumatera, Jambi, Bengkulu, Bangka Belitung, Riau Island, West Kalimantan, North Sulawesi, Central Sulawesi, Southeast Sulawesi, West Sulawesi, South Sulawesi and Gorontalo (Region 3); South Kalimantan, East Kalimantan, North Kalimantan and Central Kalimantan (Region 4) and East Nusa Tenggara, Maluku, North Maluku, Papua and West Papua (Region 5) [40].

**
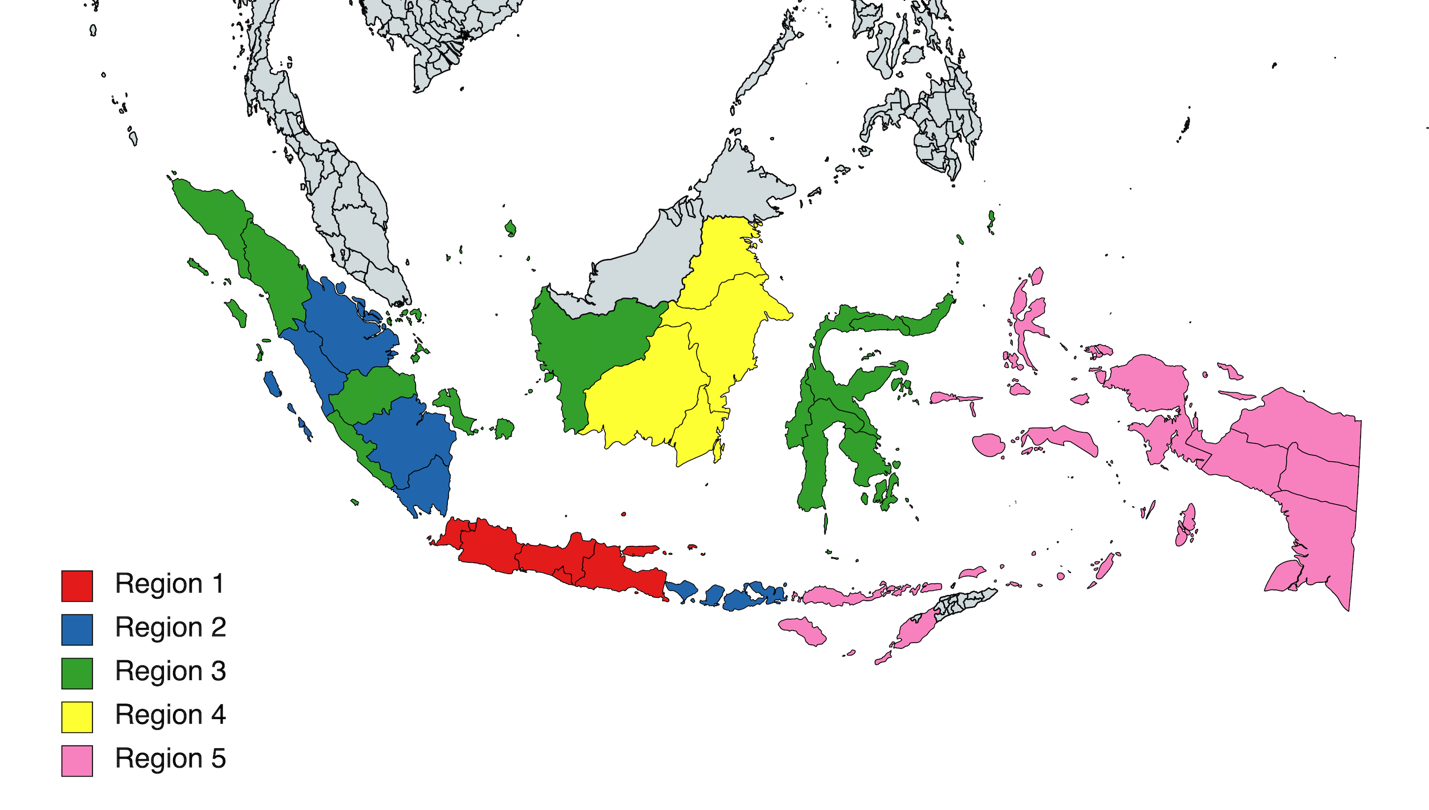
**

Figure S2 Indonesian map with 5 regions based on the national health insurance tariff group

**Table S1 List of ICD-10 codes for Noncommunicable Diseases**

| **No** | **Diagnosis (Cause)** | **ICD-10 Codes** |  |
| --- | --- | --- | --- |
| **Cardiovascular Diseases** | | | |
| 1 | Rheumatic heart disease | I01-I01.9, I02.0, I05-I09.9 |  |
| 2 | Ischemic heart disease | I20-I25.9 |  |
| 3 | Stroke | G45-G46.8, I60-I63.9, I65-I66.9, I67.0-I67.3, I67.5-I67.6, I68.1-I68.2, I69.0-I69.3 |  |
| 4 | Ischemic stroke | G45-G46.8, I63-I63.9, I65-I66.9, I67.2-I67.3, I67.5-I67.6, I69.3 |  |
| 5 | Intracerebral hemorrhage | I61-I62, I62.1-I62.9, I68.1-I68.2, I69.1-I69.2 |  |
| 6 | Subarachnoid hemorrhage | I60-I60.9, I62.0, I67.0-I67.1, I69.0 |  |
| 7 | Hypertensive heart disease | I11-I11.9 |  |
| 8 | Non-rheumatic valvular heart disease | I34-I37.8 |  |
| 9 | Non-rheumatic calcific aortic valve disease | I35-I35.9 |  |
| 10 | Non-rheumatic degenerative mitral valve disease | I34-I34.9 |  |
| 11 | Other non-rheumatic valve diseases | I36-I37.8 |  |
| 12 | Cardiomyopathy and myocarditis | B33.2, I40-I41.9, I42.1-I42.8, I43-I43.9, I51.4 |  |
| 13 | Myocarditis | B33.2, I40-I41.9, I51.4 |  |
| 14 | Alcoholic cardiomyopathy | I42.6 |  |
| 15 | Other cardiomyopathy | I42.1-I42.5, I42.7-I42.8, I43-I43.9 |  |
| 16 | Atrial fibrillation and flutter | I48-I48.9 |  |
| 17 | Aortic aneurysm | I71-I71.9 |  |
| 18 | Peripheral artery disease | I70.2-I70.8, I73-I73.9 |  |
| 19 | Endocarditis | I33-I33.9, I38-I39.9 |  |
| 20 | Other cardiovascular and circulatory diseases | I28-I28.8, I30-I31.1, I31.8-I32.8, I47-I47.9, I51.0-I51.3, I68.0, I72-I72.9, I77-I83.9, I86-I89.0, I89.9, I98, K75.1 |  |
| **NEOPLASMS** | |  |  |
| **21** | Lip and oral cavity cancer | C00-C08.9, D10.0-D10.5, D11-D11.9 |  |
| **22** | Nasopharyx cancer | C11-C11.9, D10.6 |  |
| **23** | Other pharynx cancer | C09-C10.9, C12-C13.9, D10.7 |  |
| **24** | Esophageal cancer | C15-C15.9, D00.1, D13.0 |  |
| **25** | Stomach cancer | C16-C16.9, D00.2, D13.1, D37.1 |  |
| **26** | Colon and rectum cancer | C18-C21.9, D01.0-D01.3, D12-D12.9, D37.3-D37.5 |  |
| **27** | Liver cancer | C22-C22.9, D13.4 |  |
| **28** | Gallbladder and biliary tract cancer | C23-C24.9, D13.5 |  |
| **29** | Pancreatic cancer | C25-C25.9, D13.6-D13.7 |  |
| **30** | Larynx cancer | C32-C32.9, D02.0, D14.1, D38.0 |  |
| **31** | Tracheal, bronchus, and lung cancer | C33-C34.9, D02.1-D02.3, D14.2-D14.3, D38.1 |  |
| **32** | Malignant skin melanoma | C43-C43.9, D03-D03.9, D22-D23.9, D48.5 |  |
| **33** | Non-melanoma skin cancer | C44-C44.9, D04-D04.9, D49.2 |  |
| **34** | Breast cancer | C50-C50.9, D05-D05.9, D24-D24.9, D48.6, D49.3 |  |
| **35** | Cervical cancer | C53-C53.9, D06-D06.9, D26.0 |  |
| **36** | Uterine cancer | C54-C54.9, D07.0-D07.2, D26.1-D26.9 |  |
| **37** | Ovarian cancer | C56-C56.9, D27-D27.9, D39.1 |  |
| **38** | Prostate cancer | C61-C61.9, D07.5, D29.1, D40.0 |  |
| **39** | Testicular cancer | C62-C62.9, D29.2-D29.8, D40.1-D40.8 |  |
| **40** | Kidney cancer | C64-C65.9, D30.0-D30.1, D41.0-D41.1 |  |
| **41** | Bladder cancer | C67-C67.9, D09.0, D30.3, D41.4-D41.8, D49.4 |  |
| **42** | Brain and central nervous system cancer | C70-C72.9 |  |
| **43** | Thyroid cancer | C73-C73.9, D09.3, D09.8, D34-D34.9, D44.0 |  |
| **44** | Mesothelioma | C45-C45.9 |  |
| **45** | Hodgkin lymphoma | C81-C81.9 |  |
| **46** | Non-hodgkin lymphoma | C82-C86.6, C96-C96.9 |  |
| **47** | Multiple myeloma | C88-C90.9 |  |
| **48** | Leukemia | C91-C95.9 |  |
| **49** | Other malignant cancers | C17-C17.9, C30-C31.9, C37-C38.8, C40-C41.9, C47-C4A, C51-C52.9, C57-C57.8, C58-C58.0, C60-C60.9, C63-C63.8, C66-C66.9, C68.0-C68.8, C69-C69.9, C74-C75.8, D07.4, D09.2, D13.2-D13.3, D14.0, D15-D16.9, D28.0-D28.1, D28.7, D29.0, D30.2, D30.4-D30.8, D31-D31.9, D35-D35.2, D35.5-D36, D36.1-D36.7, D37.2, D38.2-D38.5, D39.2, D39.8, D41.2-D41.3, D44.1-D44.8, D48.0-D48.4 |  |
| **50** | Other neoplasms | D32-D33.9, D35.3-D35.4, D42-D43.9, D45-D47.9, D49.6, K62.0-K62.1, K63.5, N60-N60.9, N84.0-N84.1, N87-N87.9 |  |
| **Chronic respiratory diseases** | | | |
| **51** | Chronic obstructive pulmonary diseases | J41-J44.9 |  |
| **52** | Pneumoconiosis | J60-J63.8, J65-J65.0, J92.0 |  |
| **53** | Asthma | J45-J46.9 |  |
| **54** | Interstitial lung disease and pulmonary sarcoidosis | D86-D86.2, D86.9, J84-J84.9 |  |
| **55** | Other chronic respiratory diseases | G47.3, J30-J35.9, J37-J39.9, J66-J68.9, J70, J70.8-J70.9, J82, J91-J92, J92.9 |  |
| **Digestive Diseases** | | | |
| **56** | Cirrhosis and other chronic liver diseases | B18-B18.9, I85-I85.9, I98.2, K70-K70.3, K71.7, K74-K74.9, K75.2, K75.4-K76.2, K76.4-K76.9, K77.8 |  |
| **57** | Upper digestive system diseases | K21-K21.9, K22.7, K25-K29.9, K31, K31.1-K31.6, K31.8 |  |
| **58** | Inguinal, femoral, and abdominal hernia | K40-K42.9, K44-K46.9 |  |
| **59** | Inflammatory bowel diseases | K50-K52.9, M09.1 |  |
| **60** | Vascular intestinal disorders | K55-K55.9 |  |
| **61** | Other digestive diseases | I84-I84.9, K20-K20.9, K22-K22.6, K22.8-K24, K31.0, K31.7, K38-K38.2, K57-K62, K62.2-K62.6, K62.8-K62.9, K64-K64.9, K66.8, K67, K68-K68.9, K77, K90-K90.9, K92.8, K93.8 |  |
| **Neurological Disorders** | | | |
| **62** | Alzheimer's disease and other dementias | F00-F03.9, G30-G31.1, G31.8-G31.9 |  |
| **63** | Parkinson's disease | G20-G20.9 |  |
| **64** | Idiopathic epilepsy | G40-G41.9 |  |
| **65** | Multiple sclerosis | G35-G35.9 |  |
| **66** | Motor neuron disease | G12.2-G12.9 |  |
| **67** | Other neurological disorders | G10-G12.1, G13-G13.8, G23-G24, G24.1-G25.0, G25.2-G25.3, G25.5, G25.8-G26.0, G36-G37.9, G61-G61.9, G70-G72, G72.2-G73.7, G90-G90.9, G95-G95.9, M33-M33.9 |  |
| **Mental Disorders** | | | |
| **68** | Eating disorders (anorexia,bulimia) | F50.0-F50.5 |  |
| **Substance Use Disorders** | | | |
| **69** | Alcohol use disorders | F10-F10.9, G31.2, G72.1, P04.3, Q86.0, R78.0, X45-X45.9, X65-X65.9, Y15-Y15.9 |  |
| **70** | Drug use disorder | F11-F16.9, F18-F19.9, P04.4, P96.1, R78.1-R78.5 |  |
| **Diabetes and Kidney Diseases** | | | |
| **71** | DM type 1 | E10-E10.1, E10.3-E10.9, P70.2 |  |
| **72** | DM type 2 | E11-E11.1, E11.3-E11.9 |  |
| **73** | Chronic Kidney Disease | D63.1, E10.2, E11.2, I12-I13.9, N02-N08.8, N15.0, N18-N18.9, Q61-Q62.8 |  |
| **74** | Acute glomerulonephritis | N00-N01.9 |  |
| **Skin and subcutaneous Diseases** | | | |
| **75** | Decubitus ulcer | L89-L89.9 |  |
| **Musculoskeletal Disorders** | | | |
| **76** | Rheumatoid arthritis | M05-M06.9, M08.0-M08.8 |  |
| **77** | Other musculoskeletal disorders | I27.1, I67.7, L93-L93.2, M00-M03.0, M03.2-M03.6, M07-M08, M08.9-M09.0, M09.2-M09.8, M30-M32.9, M34-M36.8, M40-M43.1, M65-M65.0, M71.0-M71.1, M80-M82.8, M86.3-M86.4, M87-M87.0, M88-M89.0, M89.5, M89.7-M89.9 |  |
| **Other noncommunicable diseases** | | | |
| **78** | Congenital birth defects | P96.0, Q00-Q07.9, Q10.4-Q18.9, Q20-Q28.9, Q30-Q36, Q37-Q45.9, Q50-Q60.6, Q63-Q86, Q86.1-Q87.8, Q89-Q89.8, Q90-Q93.9, Q95-Q99.8 |  |
| **79** | Hemoglobinopathies and hemolytic anemias | D55-D58.9, D59.1, D59.3, D59.5, D60-D61.9, D64.0 |  |
| **80** | Neural tube defects | Q00-Q01.9, Q05-Q05.9 |  |
| **81** | Congenital heart anomalies | Q20-Q28.9 |  |
| **82** | Orofacial clefts | Q35-Q36, Q37-Q37.9 |  |
| **83** | Down syndrome | Q90-Q90.9 |  |
| **84** | Other chromosomal abnormalities | Q87-Q87.8, Q91-Q93.9, Q95-Q95.9, Q97-Q97.9, Q99-Q99.8 |  |
| **85** | Congenital musculoskeletal and limb anomalies | Q65-Q79, Q79.6-Q79.9 |  |
| **86** | Urogenital congenital anomalies | P96.0, Q50-Q56.4, Q60-Q60.6, Q63-Q64.9 |  |
| **87** | Digestive congenital anomalies | Q38-Q45.9, Q79.0-Q79.5 |  |
| **88** | Other congenital birth defects | Q02-Q04.9, Q06-Q07.9, Q10.4-Q18.9, Q30-Q34.9, Q57, Q80-Q86, Q86.1-Q86.8, Q89-Q89.8 |  |
| **89** | Polycystic ovarian syndrome | E28.2 |  |
| **90** | Endometriosis | N80-N80.9 |  |
| **91** | Genital prolapse | N81-N81.9 |  |
| **92** | Thalassemias | D56-D56.9 |  |
| **93** | Sickle cell disorders | D57-D57.8 |  |
| **94** | G6PD deficiency | D55-D55.2 |  |
| **95** | Other hemoglobinopathies and hemolytic anemias | D55.3-D55.9, D58-D58.9, D59.1, D59.3, D59.5, D60-D61.9, D64.0 |  |
| **96** | Endocrine, metabolic, blood, and immune disorders | D52.1, D59.0, D59.2, D59.6, D66-D67, D68.0-D69.8, D70-D75.8, D76-D78.8, D86.8, D89-D89.3, E03-E07.1, E09-E09.9, E15.0, E16.0-E16.9, E20-E28.1, E28.3-E34.8, E36-E36.8, E65-E68, E70-E85.2, E88-E89.9, G24.0, G25.1, G25.4, G25.6-G25.7, G72.0, G93.7, G97-G97.9, I95.2-I95.3, I97-I97.9, I98.9, J70.0-J70.5, J95-J95.9, K43-K43.9, K62.7, K91-K91.9, K94-K95.8, M87.1, N14-N14.4, N65-N65.1, N99-N99.9, P96.2, P96.5, R50.2 |  |

Figure S3. Monthly hospital inpatient rates before and during COVID-19 at the national level (2016-2020)

Table S2 Difference in the mean estimate of COVID-19’s effect (IRR) on monthly visit rates by a group of diagnosis and patients with CVDs and multimorbidity by regions

| **Outcome** | **Hospital** | | **PHC** |
| --- | --- | --- | --- |
|  | **Outpatient visits**  **Mean Difference (CI)** | **Inpatient visits**  **Mean Difference (CI)** | **Outpatient visits**  **Mean Difference (CI)** |
| **Healthcare utilization among chronic disease patients in different diagnosis groups ^1^** |  |  |  |
| (1) No CVDs, but with single chronic morbidity | -0.05 (-0.26,0.16) | 0.13 (-0.03,0.29) | -0.02 (-0.11,0.07) |
| (2) No CVDs, but with multimorbidity | -0.06 (-0.22,0.10) | 0.09 (-0.07,0.25) | 0.01 (-0.07,0.09) |
| (3) CVDs, but no comorbidity. | -0.07 (-0.25,0.11) | 0.15 (-0.07,0.37) | -0.06 (-0.15,0.03) |
| (4) CVDs and one comorbidity | -0.02 (-0.20,0.16) | 0.14 (-0.07,0.35) | -0.05 (-0.13,0.03) |
| (5) CVDs and multimorbidity | - | - | - |
| **Healthcare utilization among patients with CVD and multimorbidity across different regions ^2^** |  |  |  |
| Region 1 | - | - | - |
| Region 2 | -0.06 (-0.20,0.08) | -0.02 (-0.17,0.13) | 0.02 (-0.24,0.28) |
| Region 3 | -0.03 (-0.21,0.15) | -0.03 (-0.22,0.16) | -0.07 (-0.28,0.14) |
| Region 4 | -0.18 (-0.36,0.00) | -0.19 (-0.36,0.22) | -0.16 (-0.43,0.11) |
| Region 5 | 0.06 (-0.22,0.34) | -0.08 (-0.28,0.12) | -0.11 (-0.51,0.29) |

^1^ Mean Difference in Incidence Rate Ratio (IRR) is estimated between group of diagnosis with 95% Confidence Interval, with patients belong to (5) CVD and multimorbidity as reference group.

^2^ Mean Difference in IRR is estimated between regions in patients with CVD and multimorbidity, with region 1 as reference group.


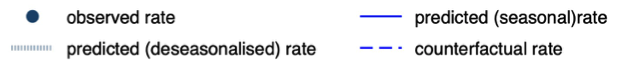


Figure S4 Monthly primary healthcare outpatient visit rates before and during COVID-19 across groups of diagnosis at the national level (2016-2020). *Interrupted Time Series Analysis in five diagnosis groups.


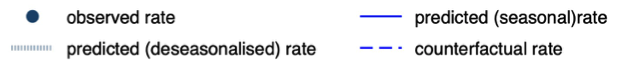


Figure S5 Monthly primary healthcare outpatient visit rates among patients with CVDs and multimorbidity before and during COVID-19 across regions in Indonesia. Interrupted time series analysis stratified by five regions in Indonesia. The regions are listed under supplementary Figure S2.

**Patient and Public Involvement**

This study did not involve patients or the public in the research design, conduct, reporting, or dissemination plans.
